# A case of neonatal tsutsugamushi disease diagnosed with the aid of rickettsial macrogenomic detection

**DOI:** 10.1186/s12887-024-04561-0

**Published:** 2024-02-02

**Authors:** Xu Yang, Ling Zhang, Shanping Chen, Wei Chen, Yushan Zhang, Yi Zhang, Jialong Liang, Ying Lv, Weiyan Wang, Yini Zhou, Rui Huang, Dongju Pan, Xueyu Li, Qiurong Li

**Affiliations:** 1 Department of Paediatrics, Pu’er People’s Hospital, Pu’er, 665000 China; 2 Department of Infectious Diseases, Pu’er People’s Hospital, Pu’er, 665000 China; 3 General Practice, Pu’er People’s Hospital, Pu’er, 665000 China; 4 Department of Anesthesiology, Pu’er People’s Hospital, Pu’er, 665000 China

**Keywords:** Tsutsugamushi disease, Macrogenetic detection, Neonates, Meningitis, rickettsial disease

## Abstract

**Background:**

Tsutsugamushi, also known as bush typhus, is a naturally occurring disease caused by *Orientia tsutsugamushi.* We reported a case of vertical mother-to-newborn transmission of *Orientia tsutsugamushi* infection in a newborn from Yunnan (China).

**Case presentation:**

Decreased fetal movements were observed at 39 weeks of gestation. After birth, the newborn (female) had recurrent fever, shortness of breath, and bruising around the mouth and extremities. At 5 h 58 min of age, the newborn was admitted for fever, shortness of breath and generalized rash. The liver was palpable 3 cm below the costal margin, and the limbs showed pitting edema. There was subcutaneous bleeding. Investigations suggested heavy infection, myocardial damage, decreased platelets. Treatment with cefotaxime and ampicillin failed. The mother was hospitalized at 29 weeks of gestation with a fever for 4 consecutive days, and an ulcerated crust was found in the popliteal fossa. Due to this pregnancy history, A diagnosis of *Orientia tsutsugamushi* infection was suspected in our index case and confirmed by macrogenomic testing and she was treated with vancomycin and meropenem, and later azithromycin for 1 week. The newborn was discharged in good general condition, gradually normalizing body temperature, and decreasing rash and jaundice. There were no abnormalities on subsequent blood macrogenomic tests for the baby. And one month later she showed good mental health, sleep, and food intake and no fever, rash, or jaundice.

**Conclusion:**

Determining the cause of symptoms is the key to treating diseases, especially the rare diseases that can be misdiagnosed.

**Suitable for people with:**

Infectious Diseases; Neonatology; Obstetrics.

## Background

Tsutsugamushi, also known as bush typhus, is a naturally occurring disease caused by *Orientia tsutsugamushi* (*Ot*), with rodents are the main source of infection and chigger mite larvae are the vector [1, 2]. Bush typhus occurs when infected mite larvae accidentally bite humans. Primarily indicated by undifferentiated febrile illnesses, the infection can be complicated and even fatal [3, 4]. The incidence of this disease is low in children [5] and even lower in newborns [6], and the clinical presentation is atypical, making it easy to miss and misdiagnose life-threatening cases [7]. In this paper, we report a case of vertical mother-to-newborn transmission of *Ot* infection in a newborn from Yunnan who was admitted to the hospital with recurrent fever, diffuse intravascular coagulation, septicemia, septic meningitis, and leakage syndrome. The diagnosis was confirmed by the detection of *Ot* by macrogenomic DNA testing. The newborn was discharged after azithromycin treatment.

## Case presentation

### General information

The newborn was a female of 5 h 58 min of age with fever, shortness of breath, and generalized rash for more than 5 h. The patient was delivered by cesarean section at Simao District People’s Hospital at 39 weeks of gestation due to fetal distress. There was no significant family history of diseases. The birth weight was 3.11 kg, amniotic fluid III degree, fecal staining, 1-min Apgar score of 8, 5-min score of 9, 10-min score of 10, postnatal recurrent fever, shortness of breath, acrals, perioral bruising, subcutaneous hemorrhage, and systemic edema. C-reactive protein was 131 mg/L, and platelets were 54 × 10^9^/L. The patient was diagnosed with severe sepsis, high-risk infant, thrombocytopenia, and myocardial damage. The patient was given cefoperazone sulbactam, vitamin C, and other symptomatic treatments, but the condition did not improve. On 18 October 2022, the newborn was transferred to the Neonatology Department of Pu’er People’s Hospital for further consultation and treatment.

### Investigations

**Fig. 1:**
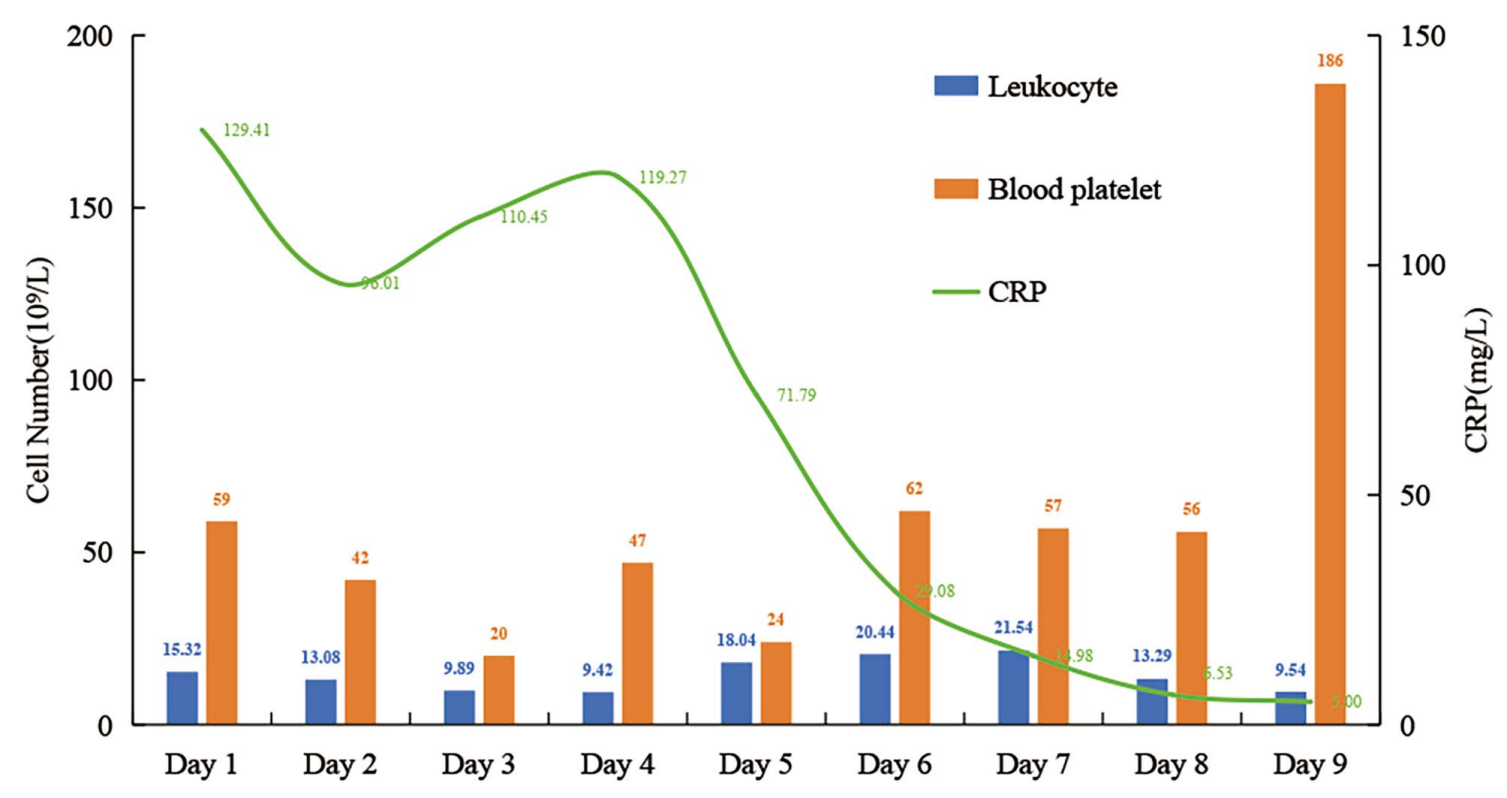
Leukocytes, blood platelets, C-reactive protein

**Fig. 2:**
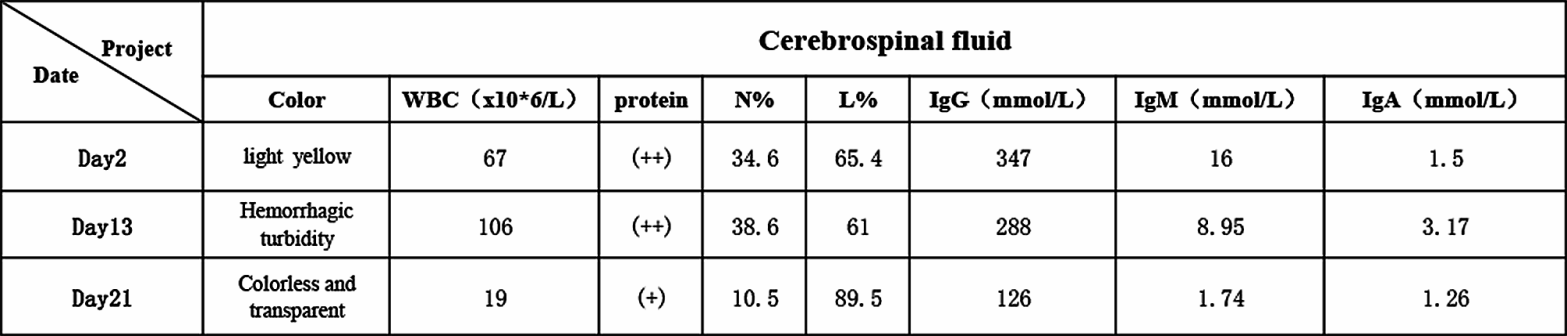
Cerebrospinal fluid laboratory indicators

On admission, the temperature was 37.6 °C, pulse rate was 149(beat/min), respiration rate was 56(breath/min), blood pressure was 60/32 mmHg, saturation was 95%, weight was 3060 g, and length was 49 cm. The patient was conscious and responsive responsive, light-moderate yellowish staining of the skin all over the body, bruising of the skin around the mouth and extremities, small patches of erythema in the upper eyelid of the right eye and the occipital region (both similar in appearance), and scattered red rash all over the body that did not fade when pressed. The extremities showed pitting edema with negative pathological signs. The liver was palpable 3 cm below the costal margin. Leukocytes (Fig. 1) were 9.4–21.5 × 10^12^/L, platelets (Fig. 1) were 24–62 × 10^9^/L, C-reactive protein (Fig. 1) was 6.5–129 mg/L, plasma fibrinogen was 1-3.4 g/L, plasma fibrin degradation products were 11–219 µg/mL, D-dimer was 3–59 µg/L, glutathione 12–38 was U/L, and albumin was 22–35 g/L. In the cerebrospinal fluid (Fig. 2), leukocytes were 63–87 × 10^6^/L, proteins were 2+, and glucose was 1.38–2.07 mmol/L. Cerebrospinal fluid culture, Gram staining, and antacid staining were all positive. Fertilizer ectoplasmosis was negative. The blood gas analysis, blood culture, parasites, human immunodeficiency virus antibody, syphilis spirochete, and haemophilus test were all negative. Cardiac ultrasound showed unclosed arterial ducts and unclosed foramen ovale. Chest computed tomography (CT) showed a bilateral lung infection. EEG indicated convulsions. Head CT revealed no abnormalities. Cranial magnetic resonance imaging (MRI) showed no abnormalities. Bone marrow examination showed no abnormalities. On 26 October 2022 a whole-blood macrogenomic test detected *Ot*.

### Diagnosis

The patient was diagnosed with neonatal sepsis and disseminated intravascular coagulation caused by *Ot* infection, which is complicated by congenital pneumonia, neonatal septic meningitis,, capillary leak syndrome, neonatal hypoproteinemia, and severe anemia.

### Treatment

Cefotaxime and ampicillin were given for 3 days as anti-infective treatment. The newborn was also treated with nutritional support, gamma globulin to suppress the anti-inflammatory antibody response, platelet transfusion, blood transfusion, anticoagulation, and albumin infusion as symptomatic treatment. Then, the patient received vancomycin and meropenem for 7 days, but the condition did not improve. A medical history review showed that the pregnant mother was hospitalized for 4 days with a fever at 29 weeks of gestation. An ulcerated scab in the popliteal fossa and abnormal coagulation was found, and the diagnosis of scrub typhus was confirmed. During treatment, considering the possible adverse effects of chloramphenicol and doxycycline on fetal development, azithromycin was chosen to treat rickettsial infection, and the newborn was discharged after 1 week of improvement. Since the incubation period of bush fever is 10 days on average [8], and the patient showed symptoms immediately after birth, it was considered that rickettsiae might have been transmitted to the newborn through the placenta. Therefore, oral treatment with azithromycin was given for 5 days. Then, macrogenomic DNA testing revealed rickettsiae with 2931 sequences, and 2890 *Ot* sequences were detected, accounting for 99.78%. After detection, azithromycin was given intravenously for 2 days. After treatment, the symptoms gradually improved. The lumbar puncture examination was repeated, suggesting that the cerebrospinal fluid leukocyte count increased, and the glucose was 1.38 mmol/L. In order to exclude the implantation of rickettsiae in the central nervous system, causing ventricular meningitis and hydrocephalus and resulting in serious neurological sequelae, azithromycin was given again for 7 days at 10 mg/kg/day intravenously. Whole blood (EDTA) was sent again to Kunming Children’s Hospital for rickettsial testing: negative Sanger sequencing for Hanseba 100 passages, negative qPCR for rickettsiae of Japanese spotted fever, rickettsiae of Mohs, Haru phagocytic apheresis, and rickettsiae of Puccin, negative qPCR for porcine erythrocytes, and negative qPCR for Bennet. The negative qPCR for Cox’s concomitant indicated that rickettsiae was completely killed in the newborn’s blood.

### Treatment outcome, follow-up, and regression

On 7 November 2022 the lumbar puncture was repeated, and the cerebrospinal fluid was sent for PMseq-DNA high-throughput genetic testing of pathogenic microorganisms of central nervous system infection (macrogenomics). Chloride was normal, and immunoglobulin was lower than before, suggesting the complete killing of rickettsiae in the central nervous system. The other indicators were completely back to normal. Finally, a clinical cure was achieved after 23 days. The trends of leukocytes (Fig. 1), platelets (Fig. 1), C-reactive protein (Fig. 1), and cerebrospinal fluid examination (Fig. 2) were shown in Figures.

## Discussion and conclusions

In recent years, the incidence of scrub typhus has gradually increased, and there is a certain regional and seasonal pattern. Indeed, there were cases of scrub typhus in all provinces of mainland China from 1952 to 1989 and from 2006 to 2017, among which the largest number of cases was in Guangdong Province (42,589 cases), followed by Yunnan Province (38,206 cases) [9]. Yunnan Province is in a highland mountainous region in Southwest China, with a predominantly tropical and subtropical climate, and scrub typhus is mainly found in Western and Southern Yunnan, while it is rarely reported in Northeastern Yunnan [10]. The peak incidence in Southwestern China is unimodal from July to October; the peak incidence in the southern region is from May to December, with a large unimodal distribution [11].

The incidence of *Ot* is mainly among farmers, bush workers, and children, but rarely among infants and even more rarely in the neonatal period. The clinical symptoms of neonatal infection are atypical, with no medical history and no typical signs of *Ot*, and the acute fever caused by the disease is often confused with other infectious fevers of unknown origins, such as typhoid fever, leptospirosis, dengue fever, malaria, and viral hemorrhagic fever [12]. Nevertheless, in some patients, the disease progresses dangerously and is prone to sepsis, shock, disseminated intravascular coagulation, septic meningitis, and leakage syndrome. As in the case of this newborn, the disease started dangerously with the above symptoms and progressed rapidly. It is extremely important to treat the cause of neonatal rickettsial infection, and any delay could lead to incurable multi-organ failure. This newborn was not treated with azithromycin until the mother’s medical history was obtained. The rickettsial infection could not be ruled out, and the diagnosis was confirmed by macrogenomic testing, and the intravenous azithromycin was continued. If left untreated, it could have been life-threatening and result in death.

The incubation period of scrub typhus is usually 6–20 days (average 10 days) [8], and this patient had a fever and rash immediately after birth, which is inconsistent with the common pathogenic process of scrub typhus infection. Therefore, it was inferred that the rickettsiae, in this case, were transmitted vertically to the fetus via the placenta, so the transmission route of this neonatal scrub typhus was completely different from the classical transmission route of scrub typhus.

The newborn, in this case, presented with fever, sepsis, and disseminated intravascular coagulation. Therefore, there was a need to exclude all infections through blood culture and cerebrospinal fluid culture performed after admission. The fluids were repeatedly tested by Frita Wai Bui, but all were negative, which was considered to be related to the low sensitivity of the Wai Bui test for scrub typhus [13]. In addition, current traditional laboratory diagnostic methods such as indirect immunofluorescence assay (IIFA), enzyme-linked immunosorbent assay (ELISA), and polymerase chain reaction (PCR) have limitations in clinical application due to lack of sensitivity, high variation in human factors, and high cost. Recent studies have shown that macrogenomic second-generation sequencing technology has broad pathogen coverage, requires little technical skill, is not affected by the use of antibiotics, and can accurately identify infections by *Ot* or other pathogens [14]. If this technology can be promoted on a large scale at a reduced cost, it will provide great help for accurate diagnosis and early treatment of *Ot* infection, effectively improving the survival rate and prognosis of critically ill children.

*Ot* infection is often treated with tetracyclines, chloramphenicol, or azithromycin, and most children treated with these drugs have their fever reduced within 24 h. Still, tetracyclines and doxycycline can inhibit bone development in fetuses and infants, and tetracyclines are contraindicated in children under 8 years of age. There is no significant difference in the efficacy of chloramphenicol and azithromycin in scrub typhus, and the hematological toxicity of chloramphenicol is significantly higher than that of azithromycin at therapeutic doses [15]. The use of chloramphenicol (daily dose > 100 mg/kg) in newborns and premature infants may cause grey baby syndrome due to immature liver function [16]. Azithromycin is a macrolide antibiotic widely used in newborns, has little effect on mammalian ribosomes, kills rickettsiae, and has mild adverse effects. Therefore, repeated long-term (10 mg/kg/day) was used in the treatment of this newborn under monitored conditions, and, eventually, the clinical cure was achieved as shown by macrogenomic testing review to confirm that rickettsiae had been cleared from the blood and cerebrospinal fluid. It has also been suggested that rifampicin is effective against *Ot*. Rifampicin is highly penetrating, widely distributed in the body, and can cross the blood-brain barrier well, but in view of the risk of liver damage and the lack of experience with its use in the neonatal period, it was not chosen for this study, and its efficacy against *Ot* needs to be further tested.

In conclusion, determining the cause of symptoms is the key to treating diseases, especially the rare diseases that can be misdiagnosed. Macrogenomics is a powerful tool to identify pathogens causing such rare diseases.

## Data Availability

All data generated or analyzed during this study are included in this published article.

## Abbreviations

*Ot*: *Orientia tsutsugamushi*
IIFA: Indirect immunofluorescence assay
ELISA: Enzyme-linked immunosorbent assay
PCR: Polymerase chain reaction
